# Giant appendiceal neurofibroma in von Recklinghausen’s disease: A case report and literature review

**DOI:** 10.3892/ol.2014.2498

**Published:** 2014-09-04

**Authors:** LIWEN GUO, KUIFENG HE, XIN XU, GUANGLIANG LI, ZHONGQI LI, YAXIAN XIA, XIAODONG TENG, LISONG TENG

**Affiliations:** 1 Department of Surgical Oncology, The First Affiliated Hospital, School of Medicine, Zhejiang University, Hangzhou, Zhejiang 310003, P.R. China; 2 Department of Obstetrics and Gynecology, The First Affiliated Hospital, School of Medicine, Zhejiang University, Hangzhou, Zhejiang 310003, P.R. China; 3 Department of Pathology, The First Affiliated Hospital, School of Medicine, Zhejiang University, Hangzhou, Zhejiang 310003, P.R. China

**Keywords:** neurofibromatosis type 1, von Recklinghausen’s disease, appendix, neurofibromatosis

## Abstract

A 62-year-old female with neurofibromatosis type 1 (NF1; also von Recklinghausen’s disease) was diagnosed with a giant, thick-walled tubular mass, mainly located in the right abdominal area on computed tomography, following an examination for intermittent abdominal pain and increasing abdominal distension. According to the clinical manifestations and imaging features, the giant tubular mass was considered most likely to be a dilated fallopian tube associated with infection, while the possibility of obstructed bowel loops was excluded. However, the subsequent laparotomy revealed a giant appendix, caused by a large neurofibroma in the root region of the appendix, which occluded the lumen. Neurofibroma of the appendix is extremely rare, even in patients with NF1. To the best of our knowledge, only three such cases have previously been reported in the English literature to date.

## Introduction

Neurofibromatosis type 1 (NF1), also known as von Recklinghausen’s disease, is an autosomal dominant hereditary tumor syndrome, with an incidence at birth of one in 3,000 individuals, independent of ethnicity and gender (1). NF1 is caused by heterozygous mutations in the NF1 gene on chromosome 17q11.2 (2), which result in the inactivation of the neurofibromin protein. Neurofibromin is a 220-kDa cytoplasmic protein containing a region of 300 amino acids with significant homology to domains found in guanosine triphosphatase-activating proteins. The protein functions as a tumor suppressor by regulating several intracellular processes, such as RAS/cyclic adenosine monophosphate and extracellular signal-regulated kinases/mitogen-activated protein kinase cascades (1,2). This may explain the increased incidence of malignant tumors of all types in patients with neurofibromatosis compared with the general population.

Gastrointestinal tract involvement is observed in 10–25% of patients with NF1, but only 5% are symptomatic (3), usually occurring in three main pathological forms: Hyperplasia of a submucosal or myenteric nerve plexus, periampullary neuroendocrine tumors and gastrointestinal stromal tumors (GISTs). The lesions are most often located in the jejunum, followed by the stomach, ileum, duodenum and colon. The presenting symptoms of gastrointestinal neurofibromatosis may include abdominal pain or distension, intestinal obstruction, bleeding or perforation, diarrhea, nausea and constipation (3). However, involvement of the appendix is extremely rare (4). A review of the literature revealed that only three cases of neurofibroma of the appendix have been reported previously. The present study reports the fourth case of appendix involvement in a patient with NF1. Additionally, the involved appendix was the largest recorded by The First Affiliated Hospital (School of Medicine, Zhejiang University, Hangzhou, Zhejiang, China). Written informed consent was obtained from the patient.

## Case report

**Figure 1:**
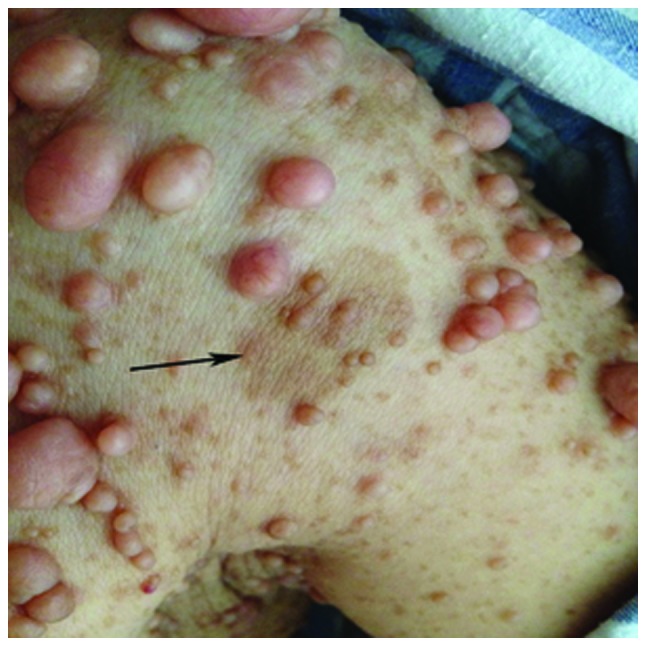
Multiple, extensive neurofibromas and café-au-lait spots (arrow) were apparent on the skin.

**Figure 2:**
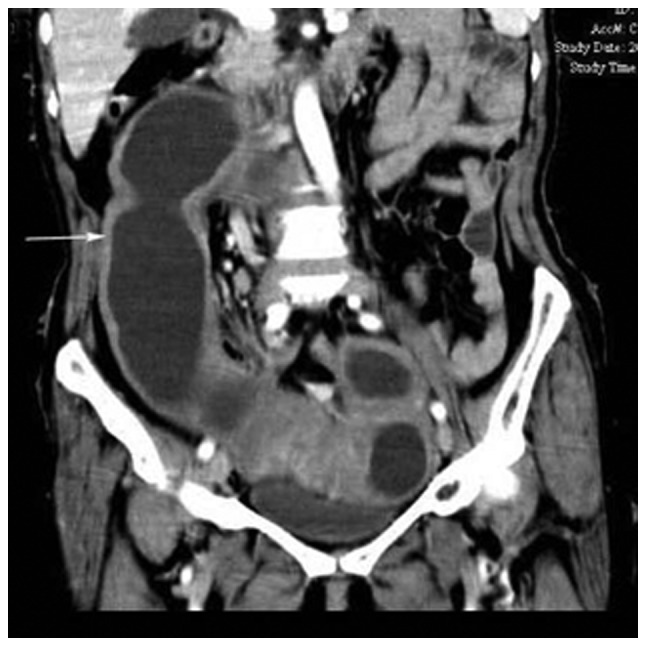
Abdominal computed tomography scan revealing a giant thick-walled tubular mass (arrow), mainly located in the right abdominal area.

A 62-year-old female with NF1 presented to The First Affiliated Hospital with intermittent lower abdominal pain, increasing abdominal distension, pelvic heaviness and tenesmus for more than one month. The patient had no history of fever, nausea or vomiting. Despite the NF1, which affected the skin, no other medical history was known, and the patient took no medication. A physical examination revealed multiple neurofibromas and café-au-lait spots on the skin (Fig. 1), and a distended abdomen with a light tender mass, ~15×10 cm in size, was palpated in the right lower quadrant. Laboratory studies were unremarkable. Contrast-enhanced computed tomography examination was performed through the abdomen, from the domes of the diaphragm to the pubic symphysis. The examination revealed a large, tubular, thick-walled mass, mainly located in the right abdominal area. The mass appeared to be filled with low-density fluid on non-contrast images, while enhanced scans suggested the wall of the mass was evenly enhanced (Fig. 2). Transvaginal color Doppler ultrasonography showed the same results.

**Figure 3:**
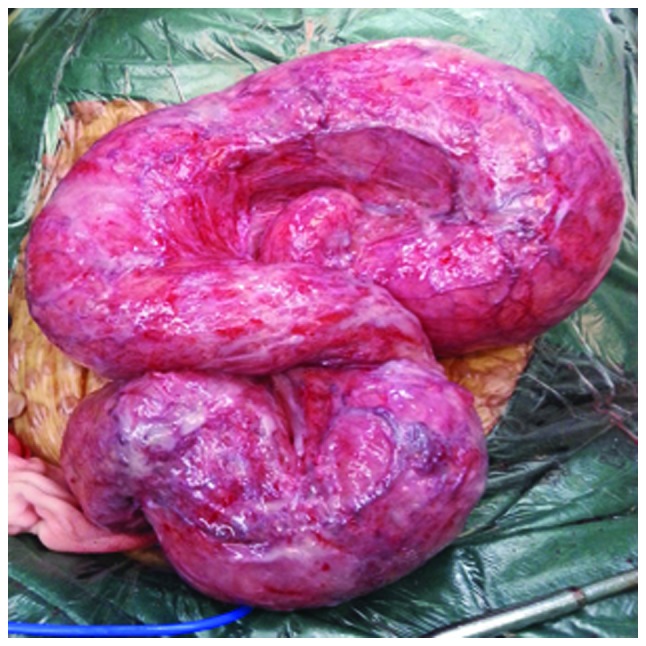
Intra-operative finding. A giant twisted appendix (38×10 cm), with varicose veins on the surface.

**Figure 4:**
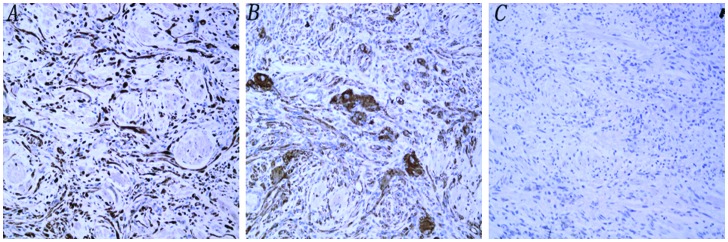
Immunohistochemical staining positive for (A) S-100 and (B) synaptophysin, and negative for (C) DOG-1 staining (all ×200 magnification).

As the clinical manifestations and imaging features did not support the diagnosis of an ileus, the giant tubular mass was considered to have originated from the fallopian tube. However, the subsequent exploratory laparotomy showed that the bilateral fallopian tubes were normal. A giant, tubular, twisted mass (38×10 cm), with varicose veins on the surface, was found in the abdominopelvic cavity (Fig. 3). A severely dilated appendix was confirmed, caused by a large submuscosal tumor, which almost blocked the lumen of the appendix. The tumor was located in the proximal portion of the appendix and extended into the distal portion along the appendiceal wall. Surgical resection of the tumor, including the right half of the colon, was performed. The post-operative recovery was normal. A pathological analysis of the specimen revealed a 9×7-cm tumor, with a submucosal and intramucosal growing pattern. The lesion contained foci of spindle cells, with a bundle-like growing pattern. Ganglion cells were also present. All surgical margins were negative and the results of the immunuohistochemistry indicated that the lesion was positive for smooth muscle actin, S100 and synaptophysin (Fig. 4). The morphological and immunohistochemical characteristics were consistent with the diagnosis of NF1. The tumor was negative for DOG-1, thus excluding a diagnosis of a GIST.

## Discussion

NF is an autosomal dominant disease with two distinct forms, NF1 and NF2. NF1, which affects 85% of patients, has complete penetrance. Due to the high susceptibility of the NF-1 gene to mutation, there can be a large variation in the clinical manifestations, even within a family. Clinically, ~50% of NF1 patients have no family history of the disorder (1). In the present case, the parents and grandparents were free of the disease, but one of the patient’s sons was affected. This phenomenon is in accordance with the genetic characteristics of NF1. Type 2, which affects 10% of patients, is associated with an abnormality of the NF2 gene, formerly called central neurofibromatosis or bilateral acoustic neurofibromatosis; however, this mainly affects the central nervous system, rather than the gastrointestinal tract (5).

NF1 is a multisystemic disorder that may affect any organ in the body. Therefore, the clinical presentations are variable and include neurofibromas, café-au-lait spots, optic gliomas, skeletal dysplasias, iris lischnodules and learning disabilities (2). Among these presentations, neurofibromas and café-au-lait spots are the most typical. Neurofibromas manifest as benign, focal cutaneous or subcutaneous lesions, or as plexiform tumors, which usually emerge in adolescence and increase in both number and size with age. These neurofibromas are usually asymptomatic. However, the unsightly appearance is a source of anguish. Café-au-lait spots are hyperpigmented lesions that may vary in color from light-brown to dark-brown, and can be observed in 95% of NF1 patients. Freckles in the axillary or inguinal regions are usually the earliest manifestations of NF1 (6).

NF1 also predisposes individuals to an increased risk of certain lesions, including neurofibromas, schwannomas, paragangliomas, duodenal carcinoid tumors, gastrointestinal autonomic nerve tumors and hyperplastic polyps (3). Gastrointestinal involvement is often asymptomatic, but when the lesions grow in size, they may present as pain, palpable abdominal masses, symptoms secondary to bowel obstruction or main vessel compression, and even as gastrointestinal bleeding when the mucosa or submucosa are involved (7,8).

Appendiceal neurofibromatosis is an extremely rare clinical entity. By reviewing the English language literature in PubMed (http://www.ncbi.nlm.nih.gov/pubmed), the present literature review found that only three such cases have been reported previously (4,9,10). Including the present case, the average age was recorded as 35.8 years, ranging between 24 and 62 years. There was no gender difference observed from the small number of cases. Among the four cases, only one was asymptomatic, which was found incidentally at cesarean section, while the remaining cases all presented with abdominal pain as the primary symptom. It is noteworthy that there was one case of solitary appendiceal neurofibromatosis, unassociated with NF1, which was even rarer. The other three cases manifested with varying degrees of skin lesions, such as café-au-lait spots and cutaneous neurofibromas. All cases had no family history of the disease (Table I).

Pathologically, the typical features of NF1 consist of a mixture of spindle cells, with wavy nuclei and strands of collagen, as well as Schwann cells, perineural fibroblasts, endothelial cells and mast cells. In addition, positivity for S100 is indicative of a neurofibroma (4). Due to the large size of the NF1 gene and the lack of mutation hot spots, mutation analysis is usually not practical as an initial tool for identifying NF-1. Therefore, the diagnosis of NF1 is mainly based on the clinical presentation. The National Institutes of Health (NIH) established a clinical diagnosis criteria in 1988 (11) as follows: At least six café-au-lait spots >5 mm in size in prepubertal individuals or >15 mm following puberty; two or more neurofibromas of any type or one plexiform neurofibroma; multiple freckles (Crowe sign) in the axillary or inguinal region; optic glioma; two or more iris hamartomas (Lisch nodules); a distinctive osseous lesion, such as sphenoid dysplasia or thinning of the long bone cortex, with or without pseudoarthrosis; and history of a first-degree relative with NF1 (12). Two or more of these signs are required for the diagnosis of NF1.

Although NF1 is a type of benign tumor, it also carries a certain degree of risk for malignant transformation, particularly in individuals >40 years old. Pain or rapid enlargement of a lesion should arouse suspicion of sarcomatous change (13). However, according to a number of studies, chemotherapy and radiotherapy are often ineffective against this tumor. Surgical treatment is aimed at alleviating the symptoms that arise when NF tumors compress nearby tissues or organs, which can consequently cause damage (14,15); for these asymptomatic patients, surgical excision remains controversial (7).

In conclusion, NF1 is a rare multisystem genetic disease, which is characterized by the growth of noncancerous tumors, termed neurofibromas and café-au-lait spots, on the skin. However, the involvement of the appendix is extremely rare. To the best of our knowledge, only three cases of neurofibroma associated with the appendix have been reported previously in the English literature. A large neurofibroma of the appendix may result in numerous complications and may exhibit the potential for malignant transformation. Therefore, timely detection and surgical removal are extremely important. The present case indicates that appendix localization must be considered in NF1 patients.

## Figures and Tables

**Table I:** Review of the literature for appendiceal neurofibromatosis.

First author/s, year (ref.)	Age/gender	Main symptom	Tumor size, cm	NF1-associated manifestation	Family history
Merck and Kindblom, 1975 (9)	24/M	Abdominal pain	NA	Skin lesions	No
Olsen, 1987 (4)	24/M	Abdominal pain	7×3	No	No
Rosenberg *et al*, 2006 (10)	33/F	Asymptomatic	12	Skin lesions	No
Present case	62/F	Abdominal pain	9×7	Skin lesions	No

NF1, neurofibromatosis type 1; M, male; F, female; NA, not available.
